# The impact of an extinction reminder on AAB renewal is sensitive to the level of association with extinction

**DOI:** 10.3758/s13420-025-00683-2

**Published:** 2025-09-08

**Authors:** A. Matías Gámez, Fátima Rojas-Iturria, Rodolfo Bernal-Gamboa

**Affiliations:** 1 Universidad de Córdoba, Córdoba, Spain; 2 Instituto Maimonides de Investigación Biomédica de Córdoba, Córdoba, Spain; 3 Universidad Nacional Autónoma de México, Mexico, Mexico; 4 Departamento de Psicología, Calle San Alberto Magno, s/n, 14071 Córdoba, Spain

**Keywords:** Context, Extinction reminder, Humans, Predictive learning, Renewal

## Abstract

An experiment using a predictive learning task with college students evaluated the impact of a stimulus associated with extinction on an AAB renewal design. Four groups of participants learned a specific relationship between two cues (X and Y) and two outcomes (O1 and O2) in Context A during the first phase. Subsequently, both cues were subjected to extinction in the same Context A. During the Test, extinction was in effect for both cues; one group experienced it in Context A (AAA), while the other three groups were tested in a second Context B. We observed a reduction in the AAB renewal effect when participants received a stimulus associated with extinction (AAB*), but not when testing involved presenting a new stimulus (AAB). However, the reductive effect of the extinction reminder was not observed when the stimulus was presented only during the 75% of the extinction trials (AAB*75). These findings suggest that, under certain circumstances, the level of association of the extinction reminder with extinction might affect its efficacy in reducing response recovery.

The recovery of an extinguished response as a consequence of changing the context between extinction and the test is usually known as the renewal effect (e.g., Bouton & Bolles, 1979), regardless of the underlying mechanisms (e.g., Nelson et al., 2011). There are three different experimental designs to study renewal. In ABA renewal, conditioning takes place in Context A, and then extinction is carried out in Context B. Conducting the test in Context A produces the recovery of the initial learning (e.g., Vila & Rosas, 2001). In ABC renewal, response recovery occurs after conditioning, and extinction and testing take place in three different contexts (e.g., Ogállar et al., 2021). Finally, in AAB renewal, the return of the original learning can be observed after conducting conditioning and extinction in the same Context A and testing the organisms in Context B (e.g., Rosas & Callejas-Aguilera, 2006). Different authors have acknowledged that renewal can be used as a laboratory model for studying the processes involved in relapse after a therapeutic intervention (e.g., Craske et al., 2018; Kimball & Kranak, 2022; Wathen & Podlesnik, 2018). All three renewal designs show that the key to response recovery (e.g., relapse) is not returning to the conditioning context but conducting testing outside the extinction context (e.g., therapist’s office). Hence, it has been proposed that the learning acquired during extinction is more sensitive to contextual changes, thereby generalizing poorly to contexts different from the one where extinction was experienced (e.g., Bouton, 2017).

One technique that seems to favor the generalization of extinction to other contexts is presenting external stimuli from extinction during the test (e.g., Bouton et al., 2006). For example, Brooks and Bouton (1994) employed an appetitive magazine-entry procedure with rats, in which a tone was paired with food in Context A during 5 days. Then, the tone was placed on extinction in Context B for 2 days. During these sessions, a light (i.e., extinction reminder) was presented several times preceding the tone. Finally, a test was conducted in Context A to assess renewal of the conditioned response (CR). The authors reported an ABA renewal effect. However, this effect was reduced when rats in the test phase received presentations of the extinction reminder (light). The effectiveness of so-called extinction reminder or extinction cues (Brooks & Bouton, 1994) in reducing the renewal effect has also been extended to an instrumental learning task with rats using food (e.g., Nieto et al., 2017, 2020; Trask & Bouton, 2016) and alcohol as reinforcers (Willcocks & McNally, 2014).

Similar evidence has also been reported with human participants (e.g., Vansteenwegen et al., 2006). For instance, Dibbets et al. (2008) using a fear conditioning task established during acquisition a conditioned-stimulus–unconditioned-stimulus relationship (CS-US) in Context A. During extinction in Context B, participants were exposed to an ampersand symbol (i.e., extinction reminder). Dibbets et al. found that presenting the extinction reminder during testing in Context A was successful in attenuating renewal (see also Dibbets & Maes, 2011). Consistent evidence on the use of extinction reminders (Gámez & Bernal-Gamboa, 2018) in renewal has been reported using predictive learning tasks (e.g., Bustamante et al., 2016), and instrumental learning preparations (e.g., Gámez & Bernal-Gamboa, 2019).

Additionally, there are reports of the effectiveness of the extinction reminders with participants relevant to applied behavior analysts, such as alcohol drinkers (Collins & Brandon, 2002; see Fisher et al., 2015, for related findings with children with problem behavior). Nevertheless, it is important to note that other studies with clinically relevant populations (spider-fearful and public-speaking-fearful participants) have reported modest or null effects when using extinction reminders (Culver et al., 2011; Dibbets et al., 2013; Laborda et al., 2016). These data do not necessarily call into question the effectiveness of using extinction reminders in some applied settings. A recent systematic review on conditioned fear renewal in humans suggests that the mixed evidence on the impact of extinction reminders indicates that there are several factors that can potentially influence the level of effectiveness (Wang et al., 2024). Following this, it is relevant to note that the previously mentioned studies showing a modest or null effect of extinction reminders share a common feature that might explain the reduced impact of such reminders on the renewal effect: the extinction reminders did not have a strong association with the extinction treatment. For example, in a study with participants fearful of public speaking, Culver et al. (2011) used three different stimuli as extinction reminders (a unique pen, a neon-green clipboard, and a white laboratory coat) during an exposure session (i.e., extinction). Nevertheless, two of those extinction reminders were also presented during other phases of the study (see also Laborda et al., 2016), which might impair the association between the extinction reminders and the extinction treatment.

Thus, the present experiment was designed to evaluate whether the level of association between the extinction reminder and the extinction phase has any impact on the ability of the extinction reminder to reduce AAB renewal in a human predictive learning task. By level of association we refer to the empirical manipulation that was done on the number of appearances of the extinction reminder during the extinction phase. Four groups of undergraduate students participated in a task in which they learned a fictitious relationship between two cues and two outcomes (X-O1; Y-O2) in Context A (e.g., a particular restaurant). Then, extinction was in effect for both cues in the same Context A. For Groups AAB* and AAB*75 extinction was accompanied by a neon picture representing someone drinking juice on the top-left area of the screen (extinction reminder). Participants in Group AAB* received the extinction reminder throughout all the extinction trials, suggesting a strong level of association with the extinction phase, whereas for Group AAB*75 the extinction reminder was presented during 75% of the extinction trials (i.e., a moderate level of association with the extinction phase). Finally, all participants were tested in a new restaurant (Context B) except for Group AAA, which received testing in the same restaurant used previously. Cue X was evaluated in the presence of the neon picture for all groups, while the neon picture was absent for cue Y. If the neon picture is acting as an extinction reminder, we should find an attenuation of renewal in Group AAB* but not in Group AAB. Furthermore, if the level of association with extinction affects the efficacy of the extinction reminder, we should find greater levels of renewal in Group AAB*75 than in Group AAB*.

## Method

### Participants

We calculated the sample size for a large effect size of 0.14 (Cohen’s *f* of 0.40). For instance, Rosas and Callejas-Aguilera (2006) found a Cohen’s *f* of 0.70 for AAB renewal in a human predictive learning task. This was obtained based on a 4 × 2 × 2 mixed analysis of variance (ANOVA) with 2 Phase (2 measurements: Last extinction trial, Renewal test), and 2 Cue (2 measurements: X, Y) as within-subjects factors, and 4 Group as a between-subjects factor (AAA, AAB, AAB*, and AAB*75), using G*Power 3.1.9.7 (Faul et al., 2007). We used an alpha of 0.05, a power of 0.80, a correlation among repeated measures of 0.5, and a nonsphericity correction of 1.

Seventy-seven (nine men) undergraduates participated in the experiment in exchange for course credit. They were between 17 and 31 years old (mean age = 19.93 years) and had no previous experience with this task. Nineteen participants were randomly assigned to each AAA, AAB, and AAB*, and 20 to AAB*75 group. Prior to participants’ arrival at the laboratory, each of the four cubicles had been preassigned the sequence corresponding to one of the four experimental groups. Upon arrival (in groups of four), participants were invited to enter the cubicle of their choice. Of course, they were unaware of the group to which they would be assigned or of the specific contingencies they were going to experience. All students gave their informed consent before beginning the experiment, being free to abandon the task at any point of the process, though none of them did. Human ethics committee at the University of Córdoba approved this study under the protocol number CEIH 21–24.

### Apparatus and stimuli

Participants were trained individually in four adjacent isolated cubicles. Each cubicle had a personal computer in which the task was presented. The procedure was implemented using the SuperLab Pro (Cedrus Corporation) software. Participants interacted with the computer using the mouse. Food items used as cues was chosen from the pool selected by García-Gutierrez and Rosas (2003). Garlic and corn were counterbalanced as Cues X and Y. Cue F was cucumber. Diarrhea and vomit were counterbalanced as outcomes. Two fictitious restaurants (The Canadian Cabin and The Swiss Cow) were counterbalanced across participants as Contexts A and B. A neon picture representing someone drinking juice was used as the extinction reminder.

### Procedure

Informed consent was obtained from participants before beginning the experiment. The instructions and all the necessary information were presented in participants’ native language (Spanish) on the computer screen. Instructions were presented in four screens using a bold black Times New Roman 22-point font against a white background. To advance the instruction screens, participants had to click on a button labeled as “next” placed on the bottom right portion of the screen. Each participant was initially asked to read the following instructions (see Gámez et al., 2022):

(Screen 1) Recent developments in food technology have led to the chemical synthesis of food. This is very advantageous as it is very low-cost and easy to both store and transport. This revolution in the food industry may solve hunger in third-world countries. (Screen 2) However, it has been detected that some foods produce gastric problems in some people. For this reason, we are interested in selecting a group of experts to identify the foods that lead to some types of illness, and how it is manifested in each case. (Screen 3) You are about to receive a selection test where you will be looking at the files of people that have ingested different foods in a specific restaurant. You will have to indicate whether gastric problems will appear. To respond you should click the option that you consider appropriate, and then click on the button that appears at the bottom corner of the screen. It is very important to respect this order, given that only your first choice will be recorded. Your response will be random at the beginning, but do not worry, as the files increase you will become an expert.

After reading the instructions, participants were required to call the experimenter, who demonstrated how to interact with the task. The demonstration was identical to an acquisition trial, with the exception that a new cue (pasta) was presented as a predictor within a restaurant that was not used again during the experiment.

Each trial began with the sentence “Loading file of*...* (a randomly chosen full name)” during 1,500 ms. Then, a screen with a restaurant picture in the background appeared. In the middle of that screen, the picture of a food was presented (garlic, corn, or cucumber), and below that food there were two 0–100 scales, one for each consequence, containing 21 small green buttons. Each button had two numbers representing 5 points in the scale (0–5, 5–10 and so on). On top of the buttons 0–5, 25–30, 55–60 and 95–100 appeared the words “none,” “little,” “quite,” and “great,” respectively, written in bold font. These labels refer to how much the participant believes that the stomach problem will occur. Participants were requested to respond by clicking on the option they considered appropriate, first for one of the outcomes, and then for the other one. Subsequently, another screen with the restaurant in the background and the name of the illness associated with that food was presented in acquisition phase, whilst in the extinction phase no outcome was presented—that is, instead of the name of the illness, the sentence “This person did not have any disease” appeared. A button appeared in the bottom corner of the screen and read “Press here to continue...”. The experiment was conducted in three phases (see Table 1).

**Table 1:** Experimental design

Group	Acquisition	Extinction	Test
AAA	A: X-O1, F- B: Y-O2, F-	A: X-, F- B: Y-, F-	A*: X- B: Y-
AAB	A: X-O1, F- B: Y-O2, F-	A: X-, F- B: Y-, F-	B*: X- A: Y-
AAB*	A: X-O1, F- B: Y-O2, F-	A*: X-, F- B*: Y-, F-	B*: X- A: Y-
AAB*75	A: X-O1, F- B: Y-O2, F-	A*: X-, F- B*: Y-, F-	B*: X- A: Y-

Contexts A and B: Different restaurants, counterbalanced. X and Y: Garlic and corn, counterbalanced. F: Cucumber. O1 and O2: diarrhea and vomit, counterbalanced. “*” stands for the extinction reminder (a neon picture representing someone drinking juice)

#### Acquisition

Each participant was trained during 12 trials in each context with Food X being followed by the outcome O1 in Context A, while Food Y was presented followed by the outcome O2 in Context B. Therefore, in the presence of X, the correct judgment refers to O1, whereas the incorrect judgment refers to O2. The reverse is true for Y. For example, if for a given participant garlic is followed by diarrhea, then the correct judgment in the presence of garlic would be the extent to which (from 0 to 100) the participant believes the patient experienced diarrhea, and the incorrect judgment would be the extent to which they believe the patient experienced vomiting. Food F was presented in both contexts with no outcome. The correct judgment for F when presented in Context A is considered to be O1 and when presented in Context B is considered to be O2. Participants in all groups received 24 trials in each context, separated in two blocks. In each block of trials, all participants received six trials of each combination X-O1 and F- in Context A, or six trials of each combination Y-O2 and F- in Context B. Trials in each block were randomly intermixed. Half of the participants received training first in Context A and then in Context B, while the other half were first trained in Context B and then in Context A. Training in each context was preceded by a screen with the sentence “Now you should analyze the files of the people that ate at restaurant... (name of the restaurant: ‘The Canadian Cabin or The Swiss Cow).”

#### Extinction

This phase began inmediately after acquisition phase. All participants received 24 extinction trials in each context, identical to the acquisition trials, except that after participants submitted their response no outcome was presented (as we said above, instead of the name of the illness, the sentence “This person did not have any disease” appeared). Cue X underwent extinction in Context A, and cue Y in Context B. Cue F was treated as in acquisition phase. In each of the extinction trials, we presented on the top-left area of the screen the extinction reminder for Group AAB*. For Group AAB*75 the extinction reminder was randomly presented in 18 trials only (75% of the extinction trials). Training in each context was preceded as well in the screen by the sentence “Now you should analyze the files of the people that ate at restaurant... (name of the restaurant).” The order of presentation of the contexts was counterbalanced across participants.

#### Test

This phase began inmediately after extinction phase. All participants received a test trial for each context with X and Y cues only. Unlike the extinction phase, in this phase no feedback was given to participants after their response. All groups were tested with the extinction reminder, which was presented only in the context in which Food X was presented (Context A in Group AAA and Context B in the rest of groups). The order of presentation of the contexts was counterbalanced across participants.

### Dependent variable and statistical analysis

Predictive judgments were requested throughout the acquisition, extinction, and test phases. Data were removed if ratings in two of the last three conditioning trials with each cue did not achieve a value of 70. This is an arbitrary value that represents approximately the third highest part of the scale and has been used previously in other studies (e.g., Balea et al., 2020). The data were analyzed using mixed ANOVA. Greenhouse–Geisser correction for the violation of the sphericity assumption was applied when appropriate. Simple effects were conducted with *t* tests or one-way ANOVAs, using corrections when appropriate (HSD Tukey or Games–Howell, depending on the variance homogeneity). As a measure of effect size, we report partial eta squared (η_p_^2^) for ANOVAs with more than one factor, eta squared (η^2^) for ANOVAs with only one factor, and Cohen’s *d* for* t* tests, and 95% confidence intervals for the effect sizes were reported for each analysis relevant to our hypotheses. Statistical analyses were performed using IBM SPSS Statistics for Windows, Version 28.0 (IBM Corp., Armonk, NY, USA). Type III sums of squares were used for all factorial ANOVAs, given the presence of interactions. Significance was set at* p* < 0.05 for all tests.

## Results

The results of the sample size calculation indicated that 24 participants were needed to achieve a power of 0.86. No volunteer who showed up was rejected, resulting in 77 participants. Following the removal criterion described above, two participants were removed from Group AAA, three from Group AAB, one was removed from Group AAB*, and four from Group AAB*75. The final group sizes were 17, 16, 19, and 16, respectively. Final *N* was 68.

**Fig. 1:**
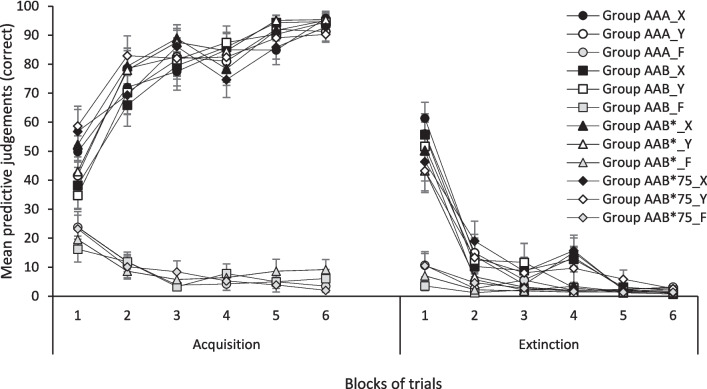
Mean correct predictive judgments for cues X, Y, and F during the six blocks of acquisition (left side) and the six blocks of extinction (right side). Error bars denote standard errors of the mean

The data from the first two phases are shown in Fig. 1, which presents the mean correct predictive judgments (those related to the expectancy of O1 when X is present, and to the expectancy of O2 when Y is present) given by participants from the four groups for Cues X and Y during six two-trial acquisition blocks (left side) and six two-trial extinction blocks (right side). Since Cue F was not counterbalanced with X and Y, we present the analysis of the data related to F separately from that of Cues X and Y.

### Correct responding to Cue F

In Fig. 1, data from Cue F have been collapsed between contexts because there were no significant differences between Cue F in Context A and Cue F in Context B. A mixed 4 (Group) × 2 (Context) × 6 (Block) ANOVA with a Greenhouse–Geisser correction conducted on the acquisition data found a main effect of Block, *F*(3.03, 194.15) = 25.95, *p* < 0.001. No other main effect or interaction was significant, largest *F* = 1.06, *p* = 0.394. Similarly, the same ANOVA conducted on the extinction data only found a main effect of Block, *F*(2.17, 139.38) = 3.59, *p* = 0.004. No other main effect or interaction was significant, largest *F* = 2.08, *p* = 0.153.

### Correct responding to Cues X and Y

#### Acquisition phase

A mixed 4 (Group) × 2 (Cue) × 6 (Block) ANOVA with a Greenhouse–Geisser correction conducted on the acquisition data found a significant main effect of Block, *F*(3.50, 224.29) = 113.39, *p* < 0.001, and a Group × Block interaction, *F*(10.51, 224.29) = 2.14, *p* = 0.020. Largest *F* for any other effect involving Group: *F* = 0.76, *p* = 0.684. Moreover, no other effect or interaction was significant, largest *F* = 1.78, *p* = 0.134.

Follow-up analyses directed to explore Group × Block interaction found that the simple effect of Group was only significant in the first block, *F*(3, 67) = 3.04, *p* = 0.035. Multiple comparisons using HSD Tukey correction only found a difference between groups AAB and AAB*75, *p* = 0.019. However, the simple effect of Block was significant for Group AAA, *F*(2.98, 47.73) = 27.02, *p* < 0.001, for Group AAB, *F*(2.95, 44.36) = 46.05, *p* < 0.001, for Group AAB*, *F*(3.27, 58.95) = 36.27, *p* < 0.001, and for Group AAB*75, *F*(2.94, 44.18) = 12.86, *p* < 0.001, suggesting that judgments were acquired similarly by all participants in most part of this phase, and that responding changed as acquisition progressed, regardless the cue presented.

#### Extinction phase

A mixed 4 (Group) × 2 (Cue) × 6 (Block) ANOVA with a Greenhouse–Geisser correction conducted on the extinction data only found a significant main effect of Block, *F*(2.94, 188.13) = 192.78, *p* < 0.001. No effect involving the Group factor was significant, largest *F* = 1.72, *p* = 0.089, nor was any other effect, largest *F* = 1.21, *p* = 0.302. That is, judgments for two cues decreased over the extinction phase, regardless of the group.

### Incorrect responding to Cues X and Y

**Fig. 2:**
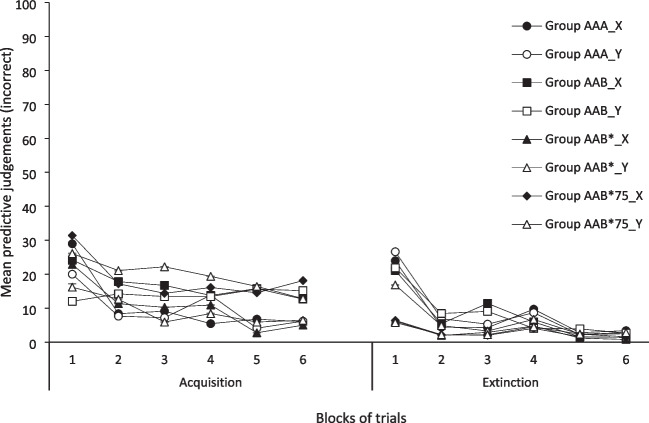
Mean incorrect predictive judgments for Cues X, and Y during the six blocks of acquisition (left side) and the six blocks of extinction (right side). Error bars denote standard errors of the mean

Figure 2 presents the mean incorrect predictive judgments (those related to the expectancy of O2 when X is present, and to the expectancy of O1 when Y is present) given by participants from the four groups for Cues X and Y during six two-trial acquisition blocks (left side) and six two-trial extinction blocks (right side).

#### Acquisition phase

A mixed 4 (Group) × 2 (Cue) × 6 (Block) ANOVA with a Greenhouse–Geisser correction conducted on the acquisition data found a significant main effect of Block, *F*(2.67, 171.06) = 14.88, *p* < 0.001, and a Cue × Block interaction, *F*(3.61, 231.04) = 3.02, *p* = 0.023. No effect involving the Group factor was significant, largest *F* = 1.19, *p* = 0.30, nor was any other effect, largest *F* = 0.89, *p* = 0.348. The analyses directed to explore the interaction found that the simple effect of Cue was only significant in the Block 1, *t*(67) = 2.71, *p* = 0.004. Cue X produced higher predictive judgments. Nevertheless, the simple effect of Block was significant in both Cues X and Y, *F*(3.21, 215.30) = 16.87, *p* < 0.001, and *F*(3.05, 204.54) = 3.66, *p* = 0.013, respectively. These results suggest again that as acquisition blocks progressed, participants learned the right relationship between each cue and the outcome.

#### Extinction phase

A mixed 4 (Group) × 2 (Cue) × 6 (Block) ANOVA with a Greenhouse–Geisser correction conducted on the extinction data found a main effect of Block, *F*(2.14, 137.43) = 21.64, *p* < 0.001, and Group, *F*(3, 64) = 2.99, *p* = 0.037. Group × Block interaction was significant as well, *F*(6.44, 137.43) = 2.36, *p* = 0.003. No other effect was significant, largest *F* = 1.13, *p* = 0.292. Follow-up analyses directed to explore the interaction found that the simple effect of Group was only significant in the Block 1, *F*(3, 67) = 3.58, *p* = 0.019. Multiple comparisons using Games–Howell correction only found a difference between groups AAA and AAB*75, *p* = 0.031. Besides, the simple effect of Block was significant for Group AAA, *F*(1.49, 23.95) = 11.74, *p* < 0.001, for Group AAB, *F*(1.97, 29.56) = 4.83, *p* = 0.016, and for Group AAB*, *F*(2.03, 36.54) = 7.94, *p* = 0.001, suggesting that, in these groups, incorrect judgments decreased during the extinction phase, regardless the cue presented. However, for Group AAB*75, there were no differences between blocks in this phase, *F* = 1.52, *p* = 0.238, indicating that incorrect predictive ratings remained low during this phase.

### Test phase

#### Correct responding to Cues X and Y

**Fig. 3:**
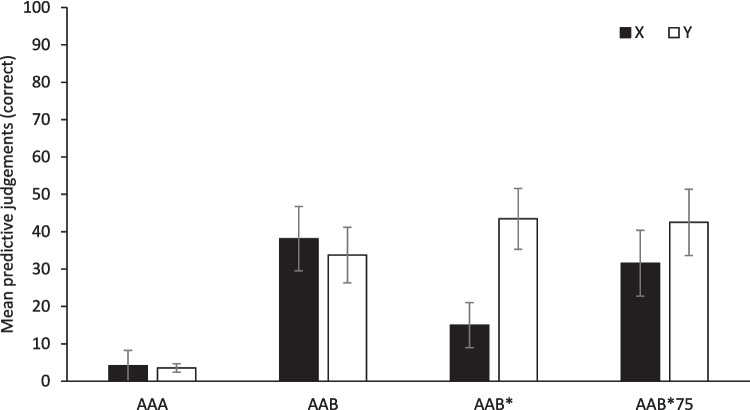
Mean correct predictive judgments for Cues X and Y during the Renewal test (Test) for all groups. Error bars denote standard errors of the mean

Figure 3 shows the mean correct predictive judgments during the Renewal test for Cues X and Y in all four groups. Differences between groups in the predictive judgments for both cues can be observed. A mixed 4 (Group) × 2 (Cue) ANOVA found a significant main effect of Group, *F*(3, 64) = 7.56, *p* < 0.001, η_p_^2^ = 0.26, and Cue, *F*(1, 64) = 4.38, *p* = 0.040, η_p_^2^ = 0.064. Moreover, a Group × Cue interaction was significant, *F*(3, 64) = 3.40, *p* = 0.023, η_p_^2^ = 0.14, 95% CI [0.00, 0.28]. A detailed analysis of this interaction showed that the simple effects of Group were significant in both Cues X and Y, *F*(3, 67) = 5.18, *p* = 0.003, η^2^ = 0.19, 95% CI [0.03, 0.33], and *F*(3, 67) = 6.93, *p* < 0.001, η^2^ = 0.24, 95% CI [0.06, 0.38], respectively. If there is a recovery of the predictive judgments due to the context change during the test, we should find higher predictive judgments in groups AAB, AAB*, and AAB*75 than in control Group AAA. Moreover, if that renewal effect is reduced or eliminated because of the presentation of a stimulus that had been previously associated with extinction, we should find no difference for Cue X between Group AAB* and Group AAA. Additionally, lower predictive judgments for Cue X than for Cue Y during the test in Group AAB* are expected. Coherently, multiple comparisons using Games–Howell correction found that, for Cue Y, there were significant differences between all three experimental groups, AAB, AAB*, and AAB*75, and the Group AAA, *p* = 0.005, *p* < 0.001, and *p* = 0.003, respectively, but there were no differences between Groups AAB and AAB*, *p* = 0.187, AAB, and AAB*75, *p* = 0.874, and AAB* and AAB*75, *p* = 1.000. However, for Cue X, there were significant differences between groups AAA and AAB, *p* = 0.006, and between Groups AAA and AAB*75, *p* = 0.033, but not between Groups AAA and AAB*, *p* = 0.320. In addition, contrary to what the figure seems to indicate, there were not significant differences between Groups AAB* and AAB, *p* = 0.077, and between Groups AAB* and AAB*75, *p* = 0.303. Finally, we only found a significant simple effect of Cue in Group AAB*, *t*(18) =  − 2.428, *p* = 0.026, *d* =  − 0.55, 95% CI [− 1.03, − 0.06]. That is, in this group predictive judgments were lower for Cue X than for Cue Y. Nevertheless, in the rest of groups there were no differences between both cues, largest *t*(15) = 1.22, *p* = 0.24.

These results suggest, first, a renewal effect of the predictive judgments for Cue Y in all groups in which there was a context change between extinction and test, and for Cue X only in groups AAB and ABB*75; and second, that presenting the extinction reminder in the test trial seemed to reduce significantly the recovery of the predictive judgments for Cue X, but only when the reminder was presented along the entire extinction phase (Group AAB*). Although we did not find a significant difference between the group AAB* and the group AAB in the response to Cue X, the presence of differences between the group AAB and the group AAA, the absence of differences between the group AAB* and the group AAA, and the observed differences in the group AAB* between Cues X and Y might be sufficient indicators of the effect of the extinction reminder in reducing the renewal effect.

#### Incorrect responding to cues X and Y

**Fig. 4:**
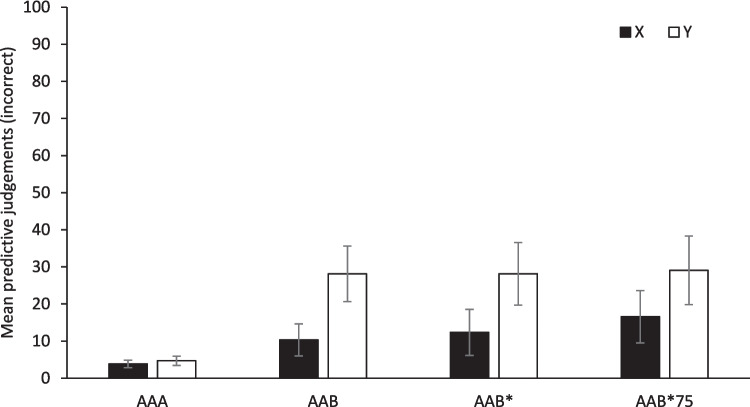
Mean incorrect predictive judgments for cues X and Y during the Renewal test (Test) for all groups. Error bars denote standard errors of the mean

Finally, Fig. 4 shows the mean incorrect predictive judgments during the Renewal test for Cues X and Y in all four groups. Although it might have been expected at this phase that incorrect predictive judgments would remain low across all four groups, regardless of the cue, differences were apparently found between the AAA group and the other groups, as well as between stimuli in at least some of the groups. Statistical analyses confirmed this observation. A mixed ANOVA, 4 (Group) × 2 (Cue) revealed that the main effects of Group and Cue were significant, *F*(3, 64) = 4.94, *p* = 0.004, and *F*(1, 64) = 5.16, *p* = 0.026, respectively, while the interaction did not reach significance, *F* < 1. Multiple comparisons using Games–Howell correction only found significant differences between Group AAA and the rest of the groups, AAB, AAB*, and AAB*75, *p* = 0.005, *p* = 0.010, and *p* = 0.007, respectively. Moreover, it is important to note that, in the Group AAA, there were no differences apparently between the incorrect judgments in the test and the last extinction block. A 2 (Cue) × 2 (Phase) repeated-measures ANOVA found that neither the main effects nor the interaction was significant, largest *F* = 2.05, *p* = 0.172. That is, changing the context during the testing also led to an increase in incorrect predictive judgments. Furthermore, although on average this increase seemed larger in the presence of Cue Y than in the presence of Cue X, when we make this comparison group by group to examine the potential effect of the extinction reminder in the incorrect predictive judgments, we find that there were no significant differences between X and Y in any group, largest *t*(15) =  − 1.81, *p* = 0.090.

## Discussion

The present experiment explored the impact of the level of association between an extinction reminder and the extinction phase on the extinction reminder’s ability to reduce AAB renewal using a predictive learning task with undergraduate students. The main observation was that testing participants in a context different from acquisition and extinction produces a recovery of responding, compared to when participants are tested in the same acquisition and extinction context. Furthermore, we observed a reduction in AAB renewal when the test was conducted in the presence of an extinction reminder that appeared throughout all extinction trials, but not when the extinction reminder was presented during 75% of the extinction trials.

The AAB renewal found in groups that received testing in Context B (i.e., groups AAB, AAB*, and AAB*75) is consistent with previous studies that also employed a within-subject design (see Rosas & Callejas-Aguilera, 2006). Additionally, the present findings extend those reported in experiments that also observed AAB renewal in predictive judgment task but that used a different retroactive interference paradigm (e.g., food first paired with diarrhea, then paired with constipation; Rosas et al., 2006; see also Rosas et al., 2001). Future studies using predictive judgment tasks might consider adding other measures that complement the responding recorded during the renewal test (e.g., gaze tracking, verbal report).

The analysis of the incorrect predictive judgments for each cue during the test phase allowed us to explore whether the reappearance of extinguished responses is due, at least in part, to a habituation process, since the decrease in response observed in extinction could reflect that a habituation process may be involved (e.g., McSweeney & Swindell, 2002). While the judgments regarding O1 in the presence of X and O2 in the presence of Y were acquired and extinguished over the first two phases of the experiment, simultaneously in those phases the judgments (or the expectancies) regarding O2 when X was present and O1 when Y was present may have been consistently habituated. Therefore, a context switch during testing may lead to an increase in the response that had been habituated (e.g., Jordan et al., 2000; Reyes-Jiménez et al., 2020). The data obtained in this study point in that direction. As noted above, in the AAA group, incorrect judgments appear to remain unchanged compared to the extinction phase. However, these judgments were significantly higher in the groups where a context change occurred between extinction and test phases, compared with the AAA group. Therefore, it could be argued that at least part of the recovery observed during the test phase, when conducted in a context different from extinction, might be due to the contextual sensitivity of habituation (see Dissegna et al., 2021, for a review). The implications of this suggestion raise theoretically relevant questions, ranging from whether what is being habituated is the response (clicking the scale) or the expectation of any outcome, to questions related to the interpretation of the renewal effect. The present findings suggest that there are two processes operating in renewal, at least in the AAB design (stimulus specificity of habituation and interference among memories). However, it has been proposed that using this design, the level of retrieval from extinction through context change could be underestimated (Rescorla, 2008), so it is important that further studies examine ABA and ABC renewal.

Although our current experimental design does not allow us to empirically address the treatment of the abovementioned ideas and possibilities, the task used in this study seems valuable for future research explicitly focused on understanding whether there are similar mechanisms in extinction and habituation and therefore between renewal and effects that impact the recovery of a habituated response (e.g., dishabituation).

Note that to the authors' knowledge, this is the first demonstration in a predictive judgment task in which an extinction reminder attenuates AAB renewal (see Bustamante et al., 2016, for similar results in ABC renewal). Furthermore, it is noteworthy that the success in attenuating renewal produced by the extinction reminder reported here is consistent with research involving instrumental learning (Gámez & Bernal-Gamboa, 2019). During acquisition, undergraduate students played a videogame in which they learned to shoot missiles at enemy ships in Context A. Then, the instrumental response underwent extinction (the shooting did not produce the destruction of any enemies) in the same Context A. Throughout this phase, participants were exposed to a red-colored rectangle on top of the screen (i.e., extinction reminder). All participants were tested in Context B. The authors found that the presentation of a red-colored rectangle during testing reduced AAB renewal compared with a control group where the red-colored rectangle was only presented during extinction. A recent meta-analysis (Alfaro et al., 2022) indicates that while there is evidence for the effectiveness of using extinction reminders in attenuating renewal, there are still few human studies, which may limit the applicability of the findings in this area to behavioral analysts interested in using behavioral strategies to reduce relapse caused by contextual changes (e.g., Kimball et al., 2023).

It is important to note that the lower levels of predictive judgments for Cue X showed by the participants in Group AAB* hardly can be viewed as an instance of a disruption or distraction provoked by the neon picture (e.g., external inhibition; Pavlov, 1927), because presenting such picture as a new stimuli during testing did not have any detectable impact on correct response performance in the AAB group. Our data suggest that the reduction effects by the neon picture are linked to its association with the extinction phase (e.g., extinction reminder). Nevertheless, the levels of predictive judgments for Cue X shown by Group AAB*75 indicate that the ability of the neon picture to reduce renewal is not only modulated by its mere association with extinction but rather relies on being strongly associated with the extinction phase. This is consistent with other reports involving human participants that showed a reduction of renewal when the extinction reminder used was strongly associated with extinction (i.e., presented at least during the 80% of the extinction trials; see Bustamante et al., 2016; Dibbets et al., 2008, 2011; Vansteenwegen et al., 2006). Thereby, the modest or null effect reported by the studies with clinically relevant populations mentioned earlier when using extinction reminder (Culver et al., 2011; Dibbets et al., 2013; Laborda et al., 2016), might be due to the lack of a high correlation between the extinction reminder and the extinction treatment. Hence, more applied research is needed in order to test this suggestion.

We proposed that the lower levels of the predictive judgments for Cue X compared with Cue Y, as shown by the participants in Group AAB*, can be explained because the neon picture is associated with extinction. However, it could be argued that participants showed higher levels of predictive judgments for Cue Y compared with Cue X because the neon picture was absent during the test of Cue Y. This explanation is unlikely, as participants in Group AAB*75 experienced testing of Cue Y without the neon picture and showed similar levels of predictive judgments for Cue X. These results suggest that presenting the neon picture in Group AAB* reduced renewal, rather than the absence of the neon picture increasing renewal.

Although our present data are consistent with an account that proposes that an extinction reminder attenuates the renewal effect, it should be noted that the present experimental design cannot provide complete clarity about the mechanism underlying the attenuation effect produced by the extinction reminder. For example, future experiments should incorporate experimental designs that offer evidence that helps to fully comprehend whether the extinction reminder functions as a conditioned inhibitor (e.g., Rescorla, 1979) or as a negative occasion setter (see Brooks & Bouton, 1994; Brooks & Bowker, 2001). It should be noted that in the present experiment, by levels of association, we refer to empirical manipulation and not to the establishment of an association, which is beyond the scope of the present experimental design. Therefore, the present experiment evaluated the level of association between the extinction reminder and the extinction phase by manipulating the percentage of presence of the extinction reminder; however, future studies might explore other ways in which such level of association is involved. For instance, participants could be shown the context and asked about the probability of the cue being present. It could also be assessed whether longer extinction reminders may have a different level of association with the extinction phase.

The experiment presented here can be added to other studies that empirically support the idea that using extinction reminders is an efficient behavioral technique to attenuate the renewal effect (e.g., Brooks & Bouton, 1994; Dibbets et al., 2008; Gámez & Bernal-Gamboa, 2019). Moreover, our results suggest that a strong association between the extinction phase and the extinction reminder might play a key role in the potential effectiveness of the extinction reminder. Since renewal is considered a laboratory model to understand the processes involved in relapse after a therapeutic intervention (e.g., Craske et al., 2018; Kimball & Kranak, 2022; Wathen & Podlesnik, 2018), our findings might be interesting in clinical and other applied contexts. They suggest that the beneficial impacts of implementing therapy reminders (extinction reminders) could be enhanced if the reminders more strongly retrieve the therapy memory.

## Data Availability

Experiment data could be available under request.

## Declarations

### Open practices statement

None of the data or materials for the experiment reported here is available. The experiment was also not preregistered. However, experiment data could be available under request.

## References

[CR1] Alfaro, F., San Martín, C., Laborda, M. A., & Míguez, G. (2022). The effect of extinction cues on response recovery: A meta-analysis. *Psykhe,**31*, 1–12. 10.7764/psykhe.2021.38063

[CR2] Balea, P., Nelson, J. B., Ogallar, P. M., Lamoureux, J. A., Aranzubia-Olasolo, M., & Sanjuan, M. d. C. (2020). Extinction contexts fail to transfer control: Implications for conditioned inhibition and occasion-setting accounts of renewal. *Journal of Experimental Psychology: Animal Learning and Cognition, 46*(4), 422–442. 10.1037/xan0000273 10.1037/xan0000273 33030954

[CR3] Bouton, M. E. (2017) Extinction: Behavioral mechanisms and their implications. In R. Menzel (Ed.), J. H. Byrne (Series ed.), *Learning theory and behavior, Vol. 1 of Learning and Memory: A Comprehensive Reference* (2nd ed., pp. 61–83). Oxford Academic.

[CR4] Bouton, M. E., & Bolles, R. C. (1979). Contextual control of the extinction of conditioned fear. *Learning and Motivation,**10*, 445–466. 10.1016/0023-9690(79)90057-2

[CR5] Bouton, M. E., Woods, A. M., Moody, E. W., Sunsay, C., & García-Gutiérrez, A. (2006). Counteracting the context-dependence of extinction: Relapse and tests of some relapse prevention methods. In M. G. Craske, D. Hermans, & D. Vansteenwegen (Eds.), *Fear and learning: Basic science to clinical application* (pp. 175–196). American Psychological Association.

[CR6] Brooks, D. C., & Bouton, M. E. (1994). A retrieval cue for extinction attenuates response recovery (renewal) caused by a return to the conditioning context. *Journal of Experimental Psychology: Animal Behavior Processes,**20*, 366–379. 10.1037/0097-7403.20.4.366 10.1037//0097-7403.19.1.77 8418218

[CR7] Brooks, D. C., & Bowker, J. L. (2001). Further evidence that conditioned inhibition is not the mechanism of an extinction cue’s effect: A reinforced prevents spontaneous recovery. *Animal Learning & Behavior,**29*, 381–388. 10.3758/BF03192903

[CR8] Bustamante, J., Uengoer, M., & Lachnit, H. (2016). Reminder cues modulate the renewal effect in human predictive learning. *Frontiers in Psychology, 7*, Article 1968. 10.3389/fpsyg.2016.01968 10.3389/fpsyg.2016.01968 PMC5167694 28066293

[CR9] Collins, B. N., & Brandon, T. H. (2002). Effects of extinction context and retrieval cues on alcohol cue reactivity among nonalcoholic drinkers. *Journal of Consulting and Clinical Psychology,**70*, 390–397. 10.1037/0022-006X.70.2.390 11952197

[CR10] Craske, M. G., Hermans, D., & Vervliet, B. (2018). State-of-the-art and future directions for extinction as a translational model for fear and anxiety. *Philosophical Transactions of the Royal Society of London. Series B, Biological Sciences, 373*(1742), 20170025. 10.1098/rstb.2017.0025 10.1098/rstb.2017.0025 PMC5790824 29352025

[CR11] Culver, N. C., Stoyanova, M., & Craske, M. G. (2011). Clinical relevance of retrieval cues for attenuating context renewal of fear. *Journal of Anxiety Disorders,**25*, 284–292. 21146358 10.1016/j.janxdis.2010.10.002

[CR12] Dibbets, P., Havermans, R., & Arntz, A. (2008). All we need is a cue to remember: The effect of an extinction cue on renewal. *Behaviour Research and Therapy,**46*, 1070–1077. 18675398 10.1016/j.brat.2008.05.007

[CR13] Dibbets, P., & Maes, J. H. R. (2011). The effect of an extinction cue on ABA-renewal: Does valence matter? *Learning and Motivation,**42*, 133–144.

[CR14] Dibbets, P., Moor, C., & Voncken, M. J. (2013). The effect of a retrieval cue on the return of spider fear. *Journal of Behavior Therapy and Experimental Psychiatry,**44*(4), 361–367. 10.1016/j.jbtep.2013.03.005 23623931 10.1016/j.jbtep.2013.03.005

[CR15] Dissegna, A., Turatto, M., & Chiandetti, C. (2021). Context-specific habituation: A review. *Animals,**11*(6), 1767. 10.3390/ani11061767 34204791 10.3390/ani11061767 PMC8231551

[CR16] Faul, F., Erdfelder, E., Lang, A. G., & Buchner, A. (2007). G*power 3: A flexible statistical power analysis program for the social, behavioral, and biomedical sciences. *Behavior Research Methods,**39*, 175–191. 10.3758/BF03193146 17695343 10.3758/bf03193146

[CR17] Fisher, W. W., Greer, B. D., Fuhrman, A. M., & Querim, A. C. (2015). Using multiple schedules during functional communication training to promote rapid transfer of treatment effects. *Journal of Applied Behavior Analysis,**48*, 713–733. 10.1002/jaba.254 26384141 10.1002/jaba.254 PMC4861165

[CR18] Gámez, A. M., & Bernal-Gamboa, R. (2018). The reinstatement effect in human predictive learning is reduced by extinction reminders. *The Spanish Journal of Psychology,**21*, 1–9. 10.1017/sjp.2018.53 10.1017/sjp.2018.53 30442214

[CR19] Gámez, A. M., & Bernal-Gamboa, R. (2019). The reoccurrence of voluntary behavior is reduced by retrieval cues from extinction. *Acta Psychologica,**200*, 102945. 10.1016/j.actpsy.2019.102945 31665622 10.1016/j.actpsy.2019.102945

[CR20] Gámez, A. M., Ogállar, P. M., & Bernal-Gamboa, R. (2022). Only a cue specifically associated with extinction reduces reinstatement in human predictive learning. *Psicológica, 43*, e14779 10.20350/digitalCSIC/14779

[CR21] García-Gutiérrez, A., & Rosas, J.M. (2003). Context change as the mechanism of reinstatement in causal learning. *Journal of Experimental Psychology: Animal Behavior Processes, 29,* 292–310. 10.1037/0097-7403.29.4.292 10.1037/0097-7403.29.4.292 14570517

[CR22] Jordan, W. P., Strasser, H. C., & McHale, L. (2000). Contextual control of long-term habituation in rats. *Journal of Experimental Psychology: Animal Behavior Processes,**26*(3), 323–339. 10.1037/0097-7403.26.3.323 10913996 10.1037//0097-7403.26.3.323

[CR23] Kimball, R. T., Greer, B. D., Fuhrman, A. M., & Lambert, J. M. (2023). Relapse and its mitigation: Toward behavioral inoculation. *Journal of Applied Behavior Analysis,**56*(2), 282–301. 10.1002/jaba.971 36715533 10.1002/jaba.971 PMC10121865

[CR24] Kimball, R. T., & Kranak, M. P. (2022). Six things practitioners should know about renewal. *Education and Treatment of Children,**45*(4), 395–410. 10.1007/s43494-022-00078-2

[CR25] Laborda, M. A., Schofield, C. A., Johnson, E. M., Schubert, J. R., George-Denn, D., Coles, M. E., & Miller, R. R. (2016). The extinction and return of fear of public speaking. *Behavior Modification,**40*(6), 901–921. 10.1177/0145445516645766 27118054 10.1177/0145445516645766

[CR26] McSweeney, F. K., & Swindell, S. (2002). Common processes may contribute to extinction and habituation. *The Journal of General Psychology,**129*(4), 364–400. 10.1080/00221300209602103 12494990 10.1080/00221300209602103

[CR27] Nelson, J. B., Sanjuan, M. d. C., Vadillo-Ruiz, S., Pérez, J., & León, S. P. (2011). Experimental renewal in human participants. *Journal of Experimental Psychology: Animal Behavior Processes, 37,* 58–70. 10.1037/a0020519 10.1037/a0020519 20822294

[CR28] Nieto, J., Mason, T. A., Bernal-Gamboa, R., & Uengoer, M. (2020). The impacts of acquisition and extinction cues on ABC renewal of voluntary behaviors. *Learning & Memory,**27*(3), 114–118. 10.1101/lm.050831.119 32071257 10.1101/lm.050831.119 PMC7029720

[CR29] Nieto, J., Uengoer, M., & Bernal-Gamboa, R. (2017). A reminder of extinction reduces relapse in an animal model of voluntary behavior. *Learning and Memory,**24*(2), 76–80. 10.1101/lm.044495.116 28096496 10.1101/lm.044495.116 PMC5238720

[CR30] Ogállar, P. M., Callejas-Aguilera, J. E., Rosas, J. M., & Lamoureux, J. A. (2021). Concurrent evidence of extinction making acquisition context specific and ABA and ABC renewal effects in human predictive learning. *Journal of Experimental Psychology: Animal Learning and Cognition,**47*(2), 137–149. 10.1037/xan0000288 34264720 10.1037/xan0000288

[CR31] Pavlov, I.P. (1927). Conditioned reflexes. Oxford University Press.

[CR32] Rescorla, R. A. (1979). Conditioned inhibition and extinction. In A. Dickinson & R. A. Boakes (Eds.), *Mechanisms of learning and motivation: A memorial volume to Jerzy Konorski* (pp. 83–110). Erlbaum.

[CR33] Rescorla, R. A. (2008). Within-subject renewal in sign tracking. *Quarterly Journal of Experimental Psychology,**61*(12), 1793–1802. 10.1080/17470210701790099 10.1080/17470210701790099 18609366

[CR34] Reyes-Jiménez, D., Iglesias-Parro, S., & Paredes-Olay, C. (2020). Contextual specificity of habituation in earthworms. *Journal of Experimental Psychology: Animal Learning and Cognition,**46*(3), 341–353. 10.1037/xan0000255 32730086 10.1037/xan0000255

[CR35] Rosas, J. M., & Callejas-Aguilera, J. E. (2006). Context switch effects on acquisition and extinction in human predictive learning. *Journal of Experimental Psychology: Learning, Memory, and Cognition,**32*, 461–474. 10.1037/0278-7393.32.3.461 16719659 10.1037/0278-7393.32.3.461

[CR36] Rosas, J. M., García-Gutiérrez, A., & Callejas-Aguilera, J. E. (2006). Effects of context change upon retrieval of first and second-learned information in human predictive learning. *Psicológica,**27*, 35–56.

[CR37] Rosas, J. M., Vila, N. J., Lugo, M., & López, L. (2001). Combined effect of context change and retention interval on interference in causality judgments. *Journal of Experimental Psychology: Animal Behavior Processes,**27*, 153–164. 10.1037/0097-7403.27.2.153 11296490

[CR38] Trask, S., & Bouton, M. E. (2016). Discriminative properties of the reinforcer can be used to attenuate the renewal of extinguished operant behavior. *Learning & Behavior,**44*, 151–161. 10.3758/s13420-015-0195-9 26400498 10.3758/s13420-015-0195-9 PMC4805475

[CR39] Vansteenwegen, D., Vervliet, B., Hermans, D., Beckers, T., Baeyens, F., & Eelen, P. (2006). Stronger renewal in human fear conditioning when tested with an acquisition retrieval cue than with an extinction retrieval cue. *Behaviour Research and Therapy,**44*, 1717–1725. 10.1016/j.brat.2005.10.014 16457776 10.1016/j.brat.2005.10.014

[CR40] Vila, N. J., & Rosas, J. M. (2001). Reinstatement of acquisition performance by the presentation of the outcome after extinction in causality judgments. *Behavioural Processes,**56*, 147–154. 10.1016/S0376-6357(01)00197-8 11738508 10.1016/s0376-6357(01)00197-8

[CR41] Wang, Y., Olsson, S., Lipp, O. V., & Ney, L. J. (2024). Renewal in human fear conditioning: A systematic review and meta-analysis. *Neuroscience and Biobehavioral Reviews,**159*, 105606. 10.1016/j.neubiorev.2024.105606 38431150 10.1016/j.neubiorev.2024.105606

[CR42] Wathen, S. N., & Podlesnik, C. A. (2018). Laboratory models of treatment relapse and mitigation techniques. *Behavior Analysis: Research and Practice,**18*(4), 362–387. 10.1037/bar0000119

[CR43] Willcocks, A. L., & McNally, G. P. (2014). An extinction retrieval cue attenuates renewal but not reacquisition of alcohol seeking. *Behavioral Neuroscience,**128*, 83–91. 10.1037/a0035595 24512068 10.1037/a0035595

